# MRI guided electrophysiological intervention with a voltage-based electro-anatomic mapping system

**DOI:** 10.1186/1532-429X-14-S1-P206

**Published:** 2012-02-01

**Authors:** Zion Tse, Charles Dumoulin, Ronald Watkins, Israel Byrd, Jeffrey Schweitzer, Raymond Kwong, Gregory F Michaud, Ehud J Schmidt

**Affiliations:** 1 Brigham and Women's Hopsital, Harvard Medical School, Boston, MA, USA; 2 University of Cincinnati College of Medicine, Cincinnati, OH, USA; 3 Standford University, Standford, CA, USA; 4 St Jude Medical, Inc., St Paul, MN, USA

## Background

MRI visualizes luminal & vessel-wall anatomy, and identifies edema & scar tissue, contributing to improved electrophysiological (EP) ablative procedures for treatment of Ventricular Tachycardia & Atrial Fibrillation. MRI-guided EP interventions will be performed for the foreseeable future partially in & outside MRI, due to the need for X-ray/Ultrasound-compliant devices. Electromagnetically tracked catheter procedures, today’s norm for most EP procedure phases; vascular navigation, Electro-Anatomic-Mapping (EAM, the diagnostic and therapeutic phases), can only be performed outside MRI. Separate MRI tracking is required in MRI, complicating EP procedures which require moving in & out of the bore [1,2]. Continuous catheter tracking using a single system would allow registration-free EAM in & outside MRI. The goal was developing an MR-compatible St. Jude Medical (SJM) EnSite NavX (ESN) voltage-based tracking [3]. ESN applies 5.8/8.0 kHz voltage bursts between 3 pairs of electrodes on the chest, detecting a catheter’s position [4], so a challenge for intra-MRI use is MR gradient ramps which interfere with ESN operation. Minimal MR Image Quality (IQ) reduction also needs to be insured, as well as <2oC patient-skin heating due to components in MRI.

## Methods

**Figure 1:**
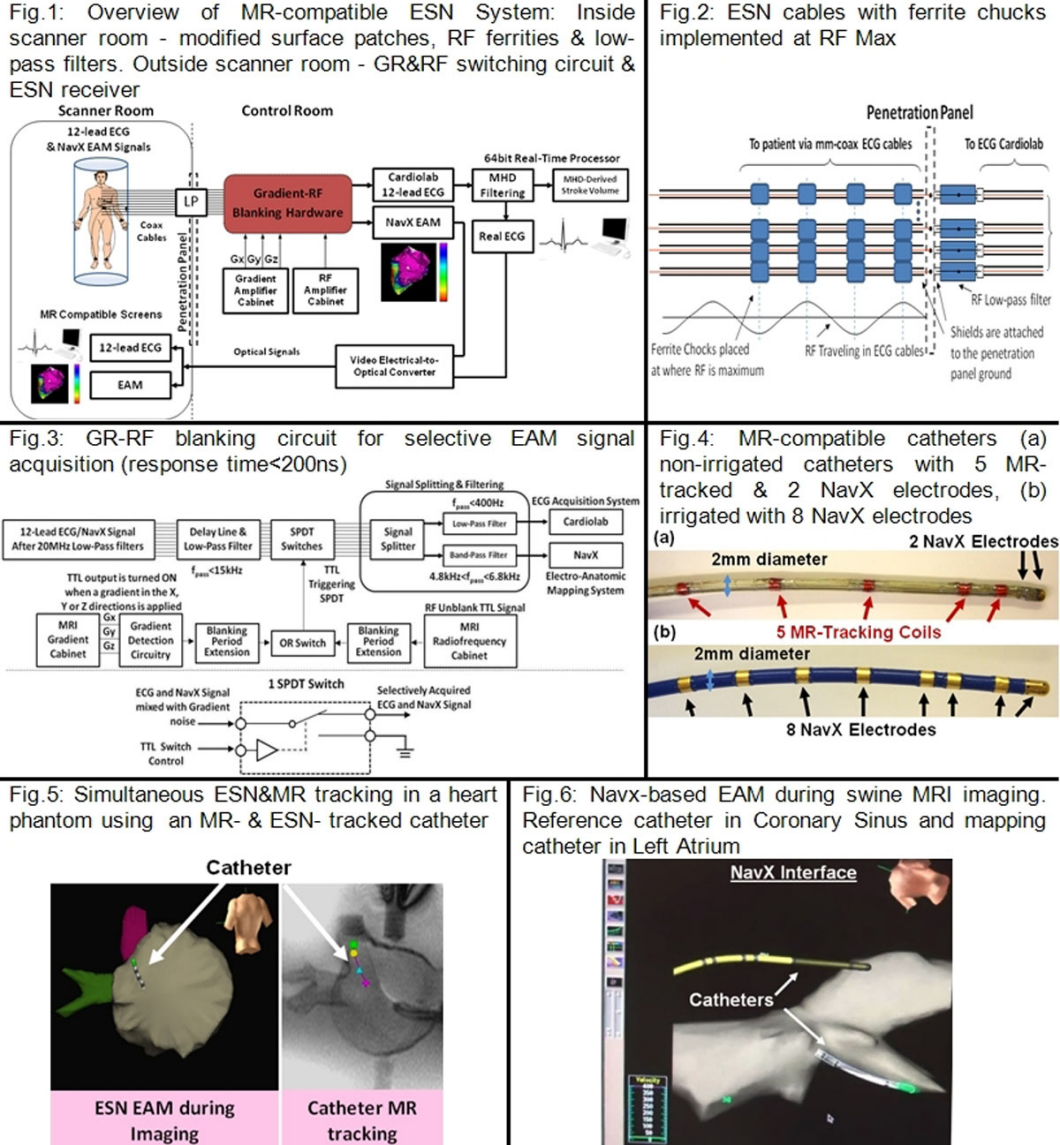


An MR-compatible ESN (Fig.1) minimized electrode heating and IQ reduction inside the scanner room with modified ESN surface-patches, ferrites on coaxial ESN leads (Fig.1(2)) , and RF filters at the penetration panel. Outside the scanner room, an electronic switching circuit, triggered by a sequence’s gradient-ramps and radio-frequency-pulse transmission (GR&RF), disconnected the ESN leads from the ESN receiver when GR&RF was detected, preventing noise from reaching it (Fig.1(3)). 2 SJM MR-compatible deflectable EP catheters were used (Fig.1(4)) [5]. The ESN was tested at 1.5T in heart phantoms and swine models with varying GRE, SSFP & FSE imaging parameters and slice orientations.

## Results

Fig.1(5) shows simultaneous ESN & MR tracking of the dual ESN&MR-tracked catheter in the phantom, verifying the lack of interference between the 2 methods. 3 EAM & MRI imaging experiments were performed in swine, also equipped with sensors to measure temperature at the surface ESN electrodes. Fig.1(6) shows EAM during imaging where catheter tracking of 2 catheters (reference catheter in the Coronary Sinus (CS) & mapping catheter in the Left Atrium) was performed simultaneously on the ESN , with <5% positional error of the CS catheter relative to its position outside MRI. IQ Reduction was <5% in both SE & GRE, with tracking updated during 60-80% of sequence duration. TR elongation was required in TR<4ms sequences. Electrode temperature rise was <1oC with 4 Watt/kg SAR sequences. Surface burns did not occur.

## Conclusions

An MR-compatible ESN system permits registration-free, minimal-heating, EAM for EP procedure in & outside MRI, with simultaneous imaging possible.
